# On the uncertainty of interdisciplinarity measurements due to incomplete bibliographic data

**DOI:** 10.1007/s11192-016-1842-4

**Published:** 2016-02-09

**Authors:** María del Carmen Calatrava Moreno, Thomas Auzinger, Hannes Werthner

**Affiliations:** E-Commerce Group, Institute of Software Technology and Interactive Systems, Vienna University of Technology, Vienna, Austria; Computer Graphics Group, Institute of Computer Graphics and Algorithms, Vienna University of Technology, Vienna, Austria; IST Austria, Klosterneuburg, Austria

**Keywords:** Interdisciplinarity, Rao–Stirling index, Bibliometrics, Missing data, Uncertainty, Optimization, Spanning tree

## Abstract

The accuracy of interdisciplinarity measurements is directly related to the quality of the underlying bibliographic data. Existing indicators of interdisciplinarity are not capable of reflecting the inaccuracies introduced by incorrect and incomplete records because correct and complete bibliographic data can rarely be obtained. This is the case for the Rao–Stirling index, which cannot handle references that are not categorized into disciplinary fields. We introduce a method that addresses this problem. It extends the Rao–Stirling index to acknowledge missing data by calculating its interval of uncertainty using computational optimization. The evaluation of our method indicates that the uncertainty interval is not only useful for estimating the inaccuracy of interdisciplinarity measurements, but it also delivers slightly more accurate aggregated interdisciplinarity measurements than the Rao–Stirling index.

**Electronic supplementary material:**

The online version of this article (doi:10.1007/s11192-016-1842-4) contains supplementary material, which is available to authorized users.

## Introduction

Most quantitative measures of the output of InterDisciplinary Research (IDR) rely on bibliometric methods. Since such methods are commonly used to inform policy in science and technology, they require reliable indicators and results. While analytical indicators and tools have been refined over time, their results are in most cases not precise. The accuracy of such indicators depends on the quality of the bibliographic data, which should be correct and complete. Unfortunately, the gathering of a correct and complete bibliographic dataset is a complicated task due to the fact that not all scientific publications are indexed by digital libraries. Current bibliographic databases, such as the Web of Science (WoS) or Scopus, do not cover books, book chapters and many regional non-English journals in which some fields mainly publish. Even conference proceedings, which constitute the main publication venues in many applied fast-changing fields, are often not indexed. The gathering and comparison of records gathered from different bibliographic sources mitigates this problem to some extent. However, an additional problem affects top–down approaches to measure IDR such as the Rao–Stirling diversity index: the need for a predefined taxonomy of disciplines that classifies all publications in the dataset. This problem cannot be solved with the comparison of data gathered from different sources because not all libraries classify their publications into a taxonomy of disciplines nor use the same taxonomy, and even those that use a taxonomy might not classify all their indexed publications with it—as is the case of WoS. Manual classification of publications into disciplinary fields is also not viable for a large number of uncategorized publications. In consequence, top–down measurements of IDR usually deliver proxy results.

In this paper we acknowledge the problem of dealing with incomplete data gathered from several libraries. We focus on the problem of uncategorized publications for the measurement of IDR with the Rao–Stirling index. We choose this index because it is a well-established bibliometric indicator that requires a complete categorization of all references into disciplinary fields; however this problem has not received adequate attention in the literature. We propose a theoretical extension of the Rao–Stirling index to account for the uncertainty resulting from references that remain uncategorized.

## Background

The field of measuring IDR heavily relies on bibliometric methods and data due to the widely-held view that scientific research is disseminated via publications. Different types of approaches exist for measuring IDR, which have been accordingly endorsed for differing needs of analysis. For an extensive review of approaches, we refer to the work of Wagner et al. (2011). Among them, the most common method for measuring IDR is citation analysis, in which an exchange or integration among fields is captured via discipline-specific citations pointing to other fields. Two distinguishable strategies for measuring IDR are bottom–up and top–down. The first approach is based on clusters of articles without a predefined taxonomy of disciplines. The clustering is based on the structural relationships of a network of publications (Boyack and Klavans 2010; Chen et al. 2010; Leydesdorff 2007; Leydesdorff et al. 2013). In contrast, top–down approaches rely on a predefined taxonomy of disciplines that is used to classify publications into disciplinary fields (Leydesdorff et al. 2013; Porter and Rafols 2009; Rafols et al. 2012). While bottom–up approaches are suited for capturing emerging developments that do not fit into existing categories, the classification-based approach is useful for large-scale explorations, such as comparisons of areas of science using an extensive amount of data or the disciplinary breadth of research institutions. The latter approach is the focus of this paper.

The results of citation analyses are subject to the quality of bibliographic data in terms of completeness and accuracy. Well-established top–down methods used to analyze the number of disciplines cited by a publication or their degree of concentration such as Shannon entropy Shannon (1948) and Herfindhal index Rhoades (1993) are designed to be used with datasets with complete information, since they cannot acknowledge the degree of missing data. This is also the case of the Rao–Stirling diversity index, a more complete top–down index proposed by Porter et al. (2007), and Porter and Rafols (2009). Precise IDR measurement using these methods requires a bibliographic dataset with: (1) complete records of references, (2) a correct list of references for each publication, (3) accurate categorization of publications into disciplinary fields, and (4) the categorization of each reference into at least one discipline. The combination of such quality characteristics results in ground-truth bibliographic data, which is rarely attainable since no publication database provides adequate correctness and completeness in respect to both references and categorization into disciplinary fields.

Concerning references, verification mechanisms as discussed by van Raan (1996) are crucial to detect incomplete records of references and remove incorrect references in bibliographic sources, such as those encountered by Moed et al. (1995) and Chen et al. (2012). In regard to taxonomies of disciplines, their accuracy have been widely discussed in the literature without reaching consensus on an adequate one National Research Council (2010), Rafols and Leydesdorff (2009). In spite of its weaknesses, the list of categories provided by WoS is the most widely used (Bensman and Leydesdorff 2009; Pudovkin and Garfield 2002). The exhaustive categorization of all references within a dataset into disciplinary fields remains an open issue under-discussed in the literature. Although the important consequences of missing data in bibliographic datasets have been acknowledged in the literature (Moed et al. 1985), to our knowledge the problem of uncategorized records in top–down IDR measurement has not been properly addressed. Some bibliometric studies minimize this problem by excluding uncategorized publications from the dataset. The use of the categories of WoS implies the exclusion of all publications other than journals indexed by WoS (i.e., proceedings papers, books, technical reports) (Bjurström and Polk 2011; Carley and Porter 2011; Chen et al. 2012). Other studies account for the percentage of uncategorized publications and compute the index on the categorized references (Rafols et al. 2012; Porter and Rafols 2009). These approaches do not take into account the potential diversity of the excluded or missing data; hence interdisciplinarity is underestimated.

A method that automatizes the assignment of disciplines was implemented by Ponomarev et al. (2013) in order to categorize authors into one out of a small set of major research fields. It is based on aggregated information on the categories of the publications of the author and their references, for which disciplines are grouped into broad categories that relate to the research activity of the group of individuals. Disciplines unrelated to the research activity of the group of individuals are categorized as ‘others’. Therefore, it does not allow for the automatic assignment of specific categories loosely related to the selected major fields, which is needed to compute the Rao–Stirling index.

In the following we propose a method which acknowledges missing data and determines the associated uncertainties (see “Method” section), as well as its evaluation and discussion in the subsequent sections.

## Method

### Introduction

In this section we briefly introduce the Rao–Stirling index and present as our main theoretical contribution an extension of it that encodes the uncertainty caused by missing bibliographic data as an uncertainty interval. The Rao–Stirling index is a distance-based indicator, inspired by the Stirling index (Stirling 2007), which not only captures the variety and balance of the disciplines cited by a paper, but also their disparity using a measure of similarity between disciplines. A hypothetical document $\mathcal {D}$ and a set $\mathcal {T}$ of ${N_{\mathcal {T}}}$ disciplines will serve as an example for the following explanations. The index can be expressed as:

$$\begin{aligned} I=1-\sum _{i,j} s_{ij}~p_i~p_j \end{aligned}$$

where $p_i$ is the proportion of references of the discipline *i* in a given paper. $s_{ij}$ is a cosine measure of similarity between the disciplines i and j. It is a matrix of similarities where disciplines that are co-cited more often by the same paper are ‘closer’ than disciplines that are less frequently co-cited (Porter and Rafols 2009). It ensures low integration scores for publications citing very similar disciplines and high integration scores for publications citing very diverse disciplines. The integration score ranges from 0 to 1 (the metric can asymptotically approach this upper limit) as variety, balance, and disparity increase.

The information on the disciplines of the categorized references of $\mathcal {D}$ can be aggregated into a vector $\mathbf {c} = (c_1, c_2, \ldots , c_{N_{\mathcal {T}}})$ of reference counts per discipline. Each count $c_i$ gives the number of references of $\mathcal {D}$ that belong to the *i*-th discipline of $\mathcal {T}$. Note that a reference can already be interdisciplinary and belong to several disciplines. By denoting the number of references that are cited by $\mathcal {D}$ with ${N_\text{ref}}$, we have for the 1-norm of $\mathbf {c}$ that

$$\begin{aligned} \sum _{i = 1}^{N_{\mathcal {T}}}c_i = {{||}\mathbf {c}{||}_{1}} \ge {N_{\text{ref}}}, \end{aligned}$$

if complete bibliographical data is assumed. Each count $c_i$ corresponds to a proportion $p_i$ by the relation $p_i = \frac{c_i}{{{||}\mathbf {c}{||}_{1}}}$. The Rao–Stirling diversity $I$ is then given as

1 $$\begin{aligned} I= 1 - \sum _{ i = 1 \atop j = 1 }^{N_{\mathcal {T}}}s_{ij} \; p_i \; p_j = 1 - \frac{1}{{{||}\mathbf {c}{||}_{1}}^2} \sum _{ i = 1 \atop j = 1 }^{N_{\mathcal {T}}}s_{ij} \; c_i \; c_j = 1 - \frac{\mathbf {c} \, \mathbf {S} \, \mathbf {c}^\intercal }{{{||}\mathbf {c}{||}_{1}}^2} \end{aligned}$$

where the similarity matrix $\mathbf {S}= (s_{ij})$ encodes the distance between the different disciplines (Stirling 2007).

### Missing Data

Problems arise when the disciplines of one or more references are unknown. As a consequence, $\mathbf {c}$ cannot be determined and $I$ is not well defined. The common approach is to simply omit these references and compute the index on the references categorized with disciplines (Bjurström and Polk 2011; Carley and Porter 2011; Chen et al. 2012; Rafols et al. 2012; Porter and Rafols 2009). Depending on the counts $\mathbf {c}$ obtained from the categorized references, as well as the number of uncategorized references, the uncertainty can widely vary. For a single uncategorized reference among dozens categorized, the effect would be minor, whereas in the converse case, the uncertainty spans nearly the whole range of the index, rendering the initial estimate meaningless.

To capture the effects of missing data, we will compute the range in which the Rao–Stirling diversity $I$ can vary when the uncategorized references are assigned to (sensible) arbitrary disciplines. While this range could be determined by enumerating all possible assignments and computing $I$ for each, such an approach is computationally infeasible as it suffers from combinatorial explosion, i.e., an uncategorized reference can be assigned to ${N_{\mathcal {T}}}$ disciplines in $2^{N_{\mathcal {T}}}$ ways. Instead, we will formulate the search for an upper and lower bound on $I$ as an optimization problem. In the following, we present its basic formulation and several subsequent refinements.

### Uncertainty Estimation

Given a document $\mathcal {D}$, let us denote with $\mathbf {c}$ the reference counts per discipline for all references *categorized* into disciplinary fields. Furthermore, $\mathcal {D}$ is referencing *u* *uncategorized* documents, i.e., documents for which we have no information on their respective disciplines. We now aim to compute new sets $\mathbf {n}_-$ and $\mathbf {n}_+$ of reference counts per discipline such that all uncategorized references are assigned to one or more disciplines. Our goal is to obtain the smallest (resp. largest) possible diversity index $I_-$ (resp. $I_+$) when computed with these new counts. Formally, we can state this requirement as

2 $$\begin{aligned} \mathbf {n}_-= & {} \mathop {\hbox {arg min}}\limits _{\mathbf {n} \in \mathbb {R}^{N_{\mathcal {T}}}} \left( 1 - \frac{\mathbf {n} \, \mathbf {S} \, \mathbf {n}^\intercal }{{{||}\mathbf {n}{||}_{1}}^2} \right) \quad \text {and} \quad \mathbf {n}_+ = \mathop {\hbox {arg max}}\limits _{\mathbf {n} \in \mathbb {R}^{N_{\mathcal {T}}}} \left( 1 - \frac{\mathbf {n} \, \mathbf {S} \, \mathbf {n}^\intercal }{{{||}\mathbf {n}{||}_{1}}^2} \right) \nonumber \\&\quad {\text {subject to}} \left\{ \begin{array}{l} c_i \le n_i \le c_i + u \quad (i = 1,2,\ldots ,{N_{\mathcal {T}}}) \\ {{||}\mathbf {c}{||}_{1}} + u \le {{||}\mathbf {n}{||}_{1}} \le {{||}\mathbf {c}{||}_{1}} + {N_{\mathcal {T}}}\, u. \end{array} \right. \end{aligned}$$

In this formulation, $\mathbf {n}_-$ and $\mathbf {n}_+$ are given as those new counts $\mathbf {n}$ that minimize and maximize the Rao–Stirling diversity defined in Eq. 1. These operations are subject to two constraints that ensure that the information obtained from the categorized references—in the form of the counts $\mathbf {c}$—is respected. The first constraint requires that the new count $n_i$ for each discipline cannot decrease below $c_i$ and that each discipline may acquire up to *u* reassigned references. The last constraint indicates that we expect each uncategorized reference to be assigned to at least one discipline and at most ${N_{\mathcal {T}}}$ disciplines. The optimization problem can also be stated in terms of proportions $\mathbf {p} = \mathbf {n} / {{||}\mathbf {n}{||}_{1}}$ (see Eq. 1), which removes the normalization in the quadratic term:

3 $$\begin{aligned} \mathbf {p}_-= & {} \mathop {\hbox {arg min}}\limits _{\mathbf {p} \in \mathbb {R}^{N_{\mathcal {T}}}} \left( 1 - \mathbf {p} \, \mathbf {S} \, \mathbf {p}^\intercal \right) \quad \text {and} \quad \mathbf {p}_+ = \mathop {\hbox {arg max}}\limits _{\mathbf {p} \in \mathbb {R}^{N_{\mathcal {T}}}} \left( 1 - \mathbf {p} \, \mathbf {S} \, \mathbf {p}^\intercal \right) \nonumber \\&\quad {\text {subject to}} \left\{ \begin{array}{l} 0 \le p_i \le \frac{c_i + u}{c_j} p_j \quad (i,j = 1,2,\ldots ,{N_{\mathcal {T}}}) \\ {{||}\mathbf {p}{||}_{1}} = 1, \end{array}. \right. \end{aligned}$$

A derivation of the transformation from Eqs. 2 to 3 can be found in Appendix “Hypercube constraints”. While the formulation of the optimization problem in terms of counts $\mathbf {n}$ allows a more intuitive description of the various constraints, the formulation in terms of proportions $\mathbf {p}$ allows a more efficient computation of the solution as we show in “Computational methods” section.

### Constraint refinement

The full range of uncertainty in the Rao–Stirling diversity index regarding missing data is given as solutions to the optimization problems stated in Eqs. 2 and 3. We found, however, that such a general form considers situations that are highly unlikely to occur in real-world scenarios. In the above formulation it is possible that each uncategorized reference increases the per-discipline count of each discipline by one. This would indicate that such a reassigned reference is maximally interdisciplinary in the sense that it covers *all* disciplines. Since this is not a realistic scenario, we limit the number of disciplines that each uncategorized reference could belong to. If we assume that each uncategorized reference cannot cover more than *k* disciplines, we can represent this as an additional constraint in optimization problem Eq. 2:

4 $$\begin{aligned} {{||}\mathbf {n}{||}_{1}} \le {{||}\mathbf {c}{||}_{1}} + k \, u. \end{aligned}$$

In proportion space, the equivalent constraint for Eq. 3 is given as

5 $$\begin{aligned} p_i \ge \frac{c_i}{{{||}\mathbf {c}{||}_{1}} + k \, u} \quad (i = 1,\ldots ,{N_{\mathcal {T}}}). \end{aligned}$$

Details on this derivation can be found in Appendix “Constraint refinement”. In “Computation of the Rao–Stirling index and its uncertainty interval” section we derive a value of $k=4$ as suitable for uncertainty computations in our context. The impact of this choice on the actual calculations is discussed in “Computational methods” section.

### Discipline pruning

A reassignment of an uncategorized reference to an arbitrary subset of disciplines can lead to highly improbable results even when the cardinality of the subset is bounded as described in “Constraint refinement” section. This arises naturally due to the maximization of the Rao–Stirling diversity index in the aforementioned optimization problems. A concrete example could be a document in the field of *computer science* that exclusively cites previous works from its own discipline but has two uncategorized references. A possible reassignment that would significantly increase its diversity can be realized by assigning them to the unrelated disciplines of, for example, *zoology* and *slavic literature*. While such an assignment is not invalid per-se, it is nevertheless prohibitively unlikely and in this section we present a method to exclude such improbable disciplines.

Our primary goal is to choose for each document a subset ${\mathcal {T}}_{\text {prune}}$ from the set $\mathcal {T}$ of all disciplines that includes such exceedingly unlikely candidates. Since we do not possess any knowledge on the disciplines of uncategorized references, we will infer this information from the disciplines of the categorized references. In the end, these deductions will lead to additional constraints for the optimization problems Eqs. 2 and 3 of the form

6 $$\begin{aligned} n_i = 0 \quad \text {respective} \quad p_i = 0 \quad \left( i \in {\mathcal {I}}_{\text {prune}}\right) \end{aligned}$$

where ${\mathcal {I}}_{\text {prune}}$ denotes the indices that correspond to the pruned disciplines that are contained in ${\mathcal {T}}_{\text {prune}}$.

A simple straightforward solution would be to just eliminate all disciplines that are not already observed from the categorized references, i.e., to set the constraint $n_i = 0$ (resp. $p_i = 0$), if $c_i = 0$. The problem with this approach is that it does not allow for the introduction of new disciplines through the reassignment of uncategorized references, which would underestimate the achievable diversity significantly.

In contrast, we take the mutual similarities of different disciplines into account for which we utilize the similarity matrix $\mathbf {S}$ as given in Eq. 1. If the categorized references are from closely related disciplines, we only permit very similar disciplines to participate in the reassignment procedure, whereas we allow a larger set of disciplines for categorized references belonging to a diverse set of disciplines.

Our method is based on the concept of a *discipline neighborhood* $\mathcal {H}_i$ of a discipline $\tau _i \in {\mathcal {T}}$ with index *i* given by all those disciplines that have a similarity higher than a given value $\Delta$, i.e.,

7 $$\begin{aligned} {\mathcal {H}}_i = \{\tau _j \in {\mathcal {T}}: \mathbf {S}_{ij} \ge \Delta \} \end{aligned}$$

where $\Delta$ effectively controls the size of $\mathcal {H}_i$. The set of permissible disciplines ${\mathcal {T}}_{\text {valid}}$ is then given as a union of such neighborhoods—one for each discipline that is observed from the categorized references. Note that the set of removed disciplines ${\mathcal {T}}_{\text {prune}}$ is given as the complement of this set, i.e., ${\mathcal {T}}_{\text {prune}}= {\mathcal {T}}\setminus {\mathcal {T}}_{\text {valid}}$. For the actual computation of this set of neighborhoods, we propose the following objectives:

*Completeness*: Each neighborhood should contain at least two observed disciplines. This ensures that each neighborhood includes at least all disciplines that are more similar than the next most similar known discipline.
*Cohesion*: The neighborhoods should form a single connected component to avoid having multiple disjoint discipline clusters. For documents with references in, for example, two dissimilar disciplines, an omission of this objective could lead to a set of permissible disciplines that are very similar to either of these two known disciplines without considering the disciplines in between them.
*Conciseness*: The neighborhoods should be chosen in such a way as to yield the smallest possible set of permissible disciplines that fulfills the previous objective. The actual meaningfulness of the upper bound of the uncertainty interval is ensured in this way.

As we show in Appendix “Discipline pruning”, we can obtain a set of permissible disciplines ${\mathcal {T}}_{\text {valid}}$ that obeys these objectives with the help of maximal spanning trees on the complete graph of disciplines when regarding the similarity matrix $\mathbf {S}$ as its adjacency matrix. Furthermore, our approach provides a user-chosen *tolerance* parameter—modulating the similarity values $\Delta$ of Eq. 7—with which the strictness of the pruning can be controlled. A tolerance of 0 would allow all disciplines to participate in the redistribution process (i.e., ${\mathcal {T}}_{\text {prune}}= \emptyset$) while a value of 1 does not introduce any additional tolerance. Note that the corresponding constraints (see Eq. 6) effectively reduce the dimensionality of the optimization problem and it is possible to compute Eqs. 2 or 3 only on those discipline counts or proportions that are not members of ${\mathcal {T}}_{\text {valid}}$. Details on the employed algorithms for these methods can be found in “Computational methods” section and our choice of the tolerance value is motivated in “Computation of the Rao–Stirling index and its uncertainty interval” section.

### Computational methods

In this section, we describe the computational methods used to compute the solutions of the optimization problems stated in Eqs. 2 or 3 while taking the constraints in Eqs. 4-6 into account. We choose different solution strategies for finding the reassignments with lowest possible diversity index $I_-$ and highest possible diversity index $I_+$. The need for different strategies lies in the nature of the similarity measure between different disciplines, given by the similarity matrix $\mathbf {S}$; it has to be *positive semidefinite* to yield a non-negative diversity index for arbitrary discipline counts. The associated quadratic form $\mathbf {c} \, \mathbf {S} \, \mathbf {c}^\intercal$ is thus a *convex* function in $\mathbf {c}$, while $- \mathbf {c} \, \mathbf {S} \, \mathbf {c}^\intercal$ is *concave*. Thus, the Rao–Stirling diversity (see Eq. 1) is a concave function and its maximization (to obtain $I_+$) can be computed with the help of quadratic programming (Nocedal and Wright 2006). Note that the constraints in Eqs. 2–5 constitute linear functions, which can be incorporated into the computation as linear equality and inequality constraints and do not impact its polynomial runtime complexity (Kozlov et al. 1980).

The minimization of a concave function has significantly worse complexity and the computation of $I_-$ lies in the class NP-hard (Pardalos and Vavasis 1991; Sahni 1974). However, we exploit the fact that the Rao–Stirling diversity is purely concave in the sense that all the eigenvalues of the similarity matrix $\mathbf {S}$ are non-positive. From this follows that all local minima lie on the vertices of the polytope that is bounded by the constraints of the optimization problems (Floudas and Visweswaran 1995). A search over all possible vertices yields the global minimum in exponential time, since the polytope for optimization problem Eq. 2 has $2^{N_{\mathcal {T}}}$ vertices, where ${N_{\mathcal {T}}}$ denotes the number of disciplines with ${N_{\mathcal {T}}}= 249$ in our case. Our constraint refinement of “Constraint refinement” section reduces the search space significantly and, apart from a more realistic uncertainty estimation, ensures the efficient computability of $I_-$. Limiting the discipline reassignment to at most four disciplines (i.e., $k = 4$) limits the search space to only $$\sum _{i=1}^{k=4} \left( {\begin{array}{c}{N_{\mathcal {T}}}\\ i\end{array}}\right) =1.6\times 10^{8}$$ vertices, which can be explored exhaustively on commodity hardware. See “Computation of the Rao–Stirling index and its uncertainty interval” section for a discussion of the choice of $k = 4$.

The discipline pruning and the corresponding maximal spanning tree have negligible computational overhead but reduce the dimensionality of the aforementioned minimization or maximization problem even further. The computation of $I_-$ especially benefits from this approach. For the minimum spanning tree computation, Prim’s algorithm is used (Prim 1957).

## Evaluation

The evaluation of the proposed method was conducted empirically. Following the framework for knowledge integration and diffusion suggested by Liu et al. (2012), the uncertainty intervals of the interdisciplinarity of the publications of a set of individuals were calculated. Ground-truth bibliographic data provided by the authors in personal interviews was used to evaluate the method. The results of our method computed with incomplete data from digital libraries were compared with the results of the Rao–Stirling index calculated with ground-truth data.

### Sample frame

The sample frame of this study consists of the publications of doctoral researchers in a Computer Science (CS) faculty of a highly ranked European university between 2009 and 2014. Doctoral researchers are usually the main authors of their publications and have a thorough knowledge of the literature they reference. We focus on CS because this field emerged as a result of integrating disciplines and it continues to be one of the most interdisciplinary fields because of its diverse applications. Moreover, CS is an ideal field to use in evaluating our method because gathering publication data with a high percentage of categorized references is especially challenging. While in other fields conferences serve as venues for community building and maintenance, in CS they focus on selectivity, quality and fast dissemination—needed in such a fast-evolving field—which drives down conference acceptance rates Grudin (2011). Therefore, CS researchers target their publications at conferences, which are regarded as the primary means of publication in the field. Since conference publications are not associated to the taxonomy of disciplines of WoS, which we use in this analysis, a high number of uncategorized references is obtained.

### Data collection

In order to gather the most complete and accurate record of publications and their references, data was gathered from different sources. First, the publication database of the university was used to collect all the publications of doctoral students of the CS faculty published between 2009 and 2014. This database contains a very exhaustive list of publications authored by those affiliated to the university, as its records are used to compute the financial assignments to the different research groups. Because the publication database of the university does not keep records of references, in the next step we gathered more data from online bibliographic databases: (1) Scopus from Elsevier, which offers high coverage of articles; and (2) WoS from Thomson Reuters, which provides a comprehensive citation search and encompasses publications of multiple online databases, resulting in multidisciplinary coverage.

The association of publications to disciplinary fields was possible using the taxonomy of disciplines of WoS, called *Category Terms* (CTs). It contains 249 CTs and is elaborated based on a combination of subject matter expert judgments and inter-journal citation patterns that together serve to cluster journals into topical groupings. Since there is no consensus on a perfect taxonomy of disciplines, the one of WoS was selected because its extensive use in the bibliometric analyses of previous related work, but other taxonomies could also be used. As a measure of similarity between CTs, we used the co-citation similarity matrix provided by Porter and Rafols (2009).

The combination of several databases increases the completeness of the record of references at the same time that it decreases the percentage of publications categorized with CTs—only journal publications indexed by WoS are categorized. Our dataset contains 1746 publications authored by 225 doctoral students. The extraction of references was possible for 1068 publications indexed by WoS or Scopus. The association of CTs to references was possible for 979 of the publications that had references indexed by WoS. A total of 12,243 references were extracted, of which 5310 are categorized with CTs.

### Computation of the Rao–Stirling index and its uncertainty interval

We calculated the Rao–Stirling index and the uncertainty interval of the 1068 publications for which the extraction of references was possible. The limit of discipline reassignment for the uncertainty interval was set to ${k=4}$. This score is at the 99th percentile of the number of CTs used by WoS to categorize the journals of our dataset. The tolerance was also set to the 99th percentile of similarity between CTs (${t=0.233}$) in order to incorporate a slight diversity into the pool of similar CTs to be used in the reassignment procedure.

**Fig. 1:**
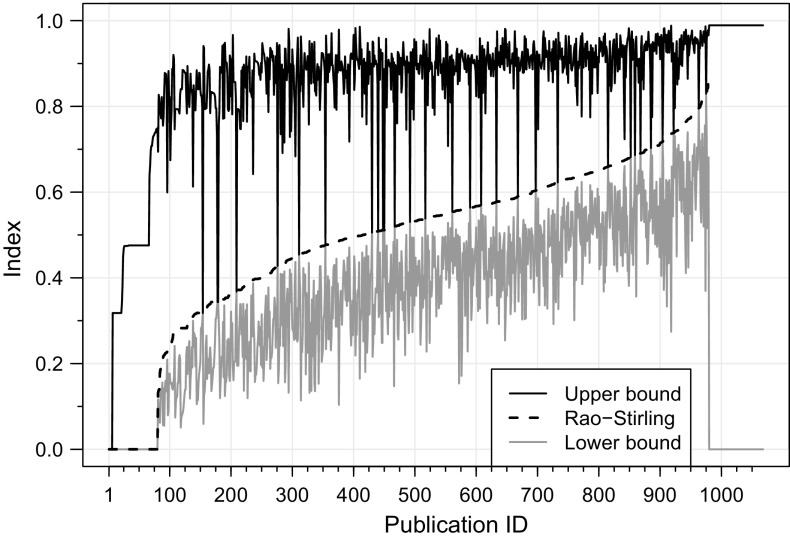
Rao–Stirling indices calculated with incomplete data (*dashed line*) and upper and lower endpoints of our uncertainty intervals (*black* and *gray solid lines*) for the 1068 students’ publications from which references could be extracted. While the Rao–Stirling index ignores the missing data, the lower and upper bounds of our uncertainty intervals take into account the uncategorized references, performing sensible reassignments of CTs that deliver the lowest and highest diversity index respectively. The publications are ordered along the x-axis according to their Rao–Stirling index

The results can be observed in Fig. 1. It is very typical for publications to have only some of their references categorized, while the rest remain uncategorized (publication IDs 81-979). When every single reference of a publication is categorized with the same single CT both endpoints of the uncertainty interval are 0, as no CTs need to be redistributed (IDs 1-6). In case where a publication that references a single CT has uncategorized references (IDs 7-80), the lower bound of the interval would be 0 (all uncategorized references could be assigned to the same single CT), while the upper bound would be greater than 0 (the uncategorized references could be assigned to different CTs). If all references of a publication are uncategorized, the Rao–Stirling cannot be computed and the size of the uncertainty interval is at maximum (IDs 980-1068).

**Fig. 2:**
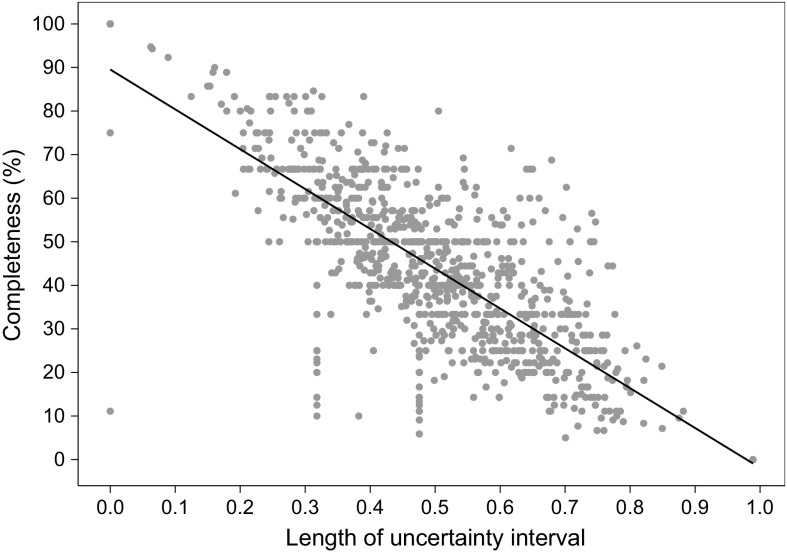
Relationship of the length of our uncertainty interval and the data completeness of the 1068 students’ publications for which references could be extracted. The completeness of a publication is defined as the ratio of categorized references in relation to its total number of references. The linear regression is represented with a *black line*. It can be seen that our approach captures the uncertainty associated with varying completeness

The size of the uncertainty interval indicates the level of accuracy of the Rao–Stirling index. The interval is large when publications contain a large proportion of uncategorized references, while it converges to a single value when all references are categorized (see Fig. 2). The significance of this relationship is confirmed through linear regression analysis with *p* value $<2.2 \times 10^{-16}$.

### Collection of ground-truth data

We refer to ground-truth data as complete and correct publication records with complete and correct categorization of references. The manual gathering of such data is very time-consuming. Therefore, a sample of publications was selected from the whole publication dataset. We applied stratified sampling with samples of equal size in each stratum, in order to obtain a sample of publications with different degrees of completeness and interdisciplinarity. Publications were divided into mutually exclusive sub-groups depending on two variables: (1) the proportion of categorized references among all references of a paper; and (2) the degree of interdisciplinarity of a publication, calculated using the Rao–Stirling index with the incomplete publication dataset that was previously gathered from the digital libraries WoS and Scopus (see “Data collection” section). Both variables were divided into 4 intervals, creating 16 sub-groups of publications. From each sub-group 3 publications were randomly selected, yielding a sample of 48 publications. First authors were invited to participate in our study. In a few cases a coauthor was invited due to reasons such as expertise or availability. In personal interviews, the participants categorized the references of their publications using one to four CTs from the taxonomy of WoS. For each interview we provided the following material:

- Digital copies of the author’s publication and all its references which were gathered manually from digital libraries.
- A print-out of the taxonomy of CTs of WoS. In order to make the search of CTs easier for the participants, CTs were grouped into macro-disciplines.

Data collection via personal interviews was chosen over a questionnaire in order to ensure the gathering of higher quality data, which allowed us to:

- Explain the importance of providing objective data. Since interdisciplinary research has a good connotation, it was important to make our participants understand that they were not going to be evaluated in terms of interdisciplinarity. We asked them to provide us with the most objective data without exaggerating interdisciplinarity or single-disciplinarity.
- Make sure that participants became acquainted with the taxonomy of CTs, as none of the participants were familiar with it.
- Confirm that participants understood their task. Participants were asked to think out loud and explain their choice of CTs for verification purposes.
- Make sure that each participant followed the same criteria to categorize publications into disciplines.

### Comparative analysis

**Fig. 3:**
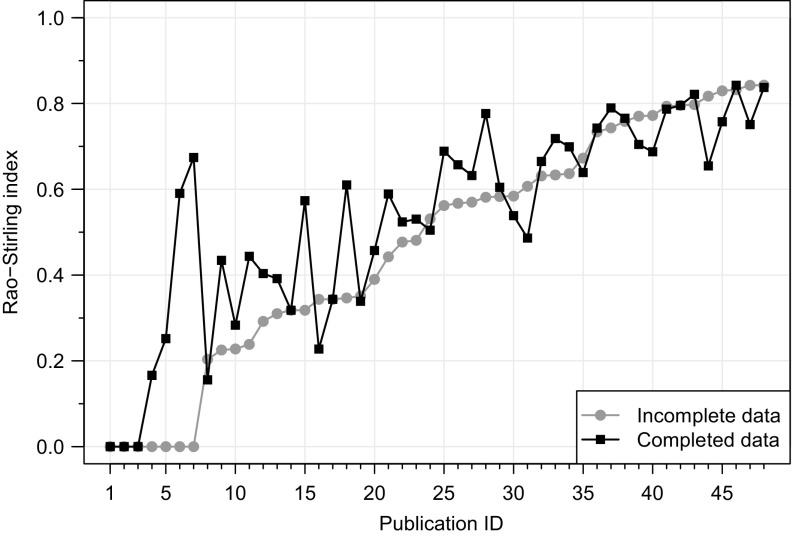
Rao–Stirling indices of the 48 publications of the sample with incomplete (*gray line*) and completed (*black line*) data. The publications are ordered according to their Rao–Stirling index with incomplete data. Depending on the degree of incompleteness, large deviations of the diversity index can be observed

In order to evaluate the performance of our method, its results were compared with the measurement of IDR based on completed data. The ground-truth data provided by the interview participants was used to complete the missing categorization of references from their publications. We computed the Rao–Stirling index of these publications again, this time using the completed data. The results of the Rao–Stirling index with completed data are compared with the results of the Rao–Stirling index with incomplete data in Fig. 3. On average the results of the Rao–Stirling index calculated with completed data are higher and less variable (see Table 1).

**Table 1:** Estimated mean and standard deviation (SD) of the Rao–Stirling index of the 48 publications of the sample calculated with incomplete and completed data. These estimated values were calculated with a bootstrapped sample of 50,000 elements with replacement

Rao–Stirling index	Estimated mean	SD
Incomplete data	0.47495	0.03929
Completed data	0.53862	0.03307

**Fig. 4:**
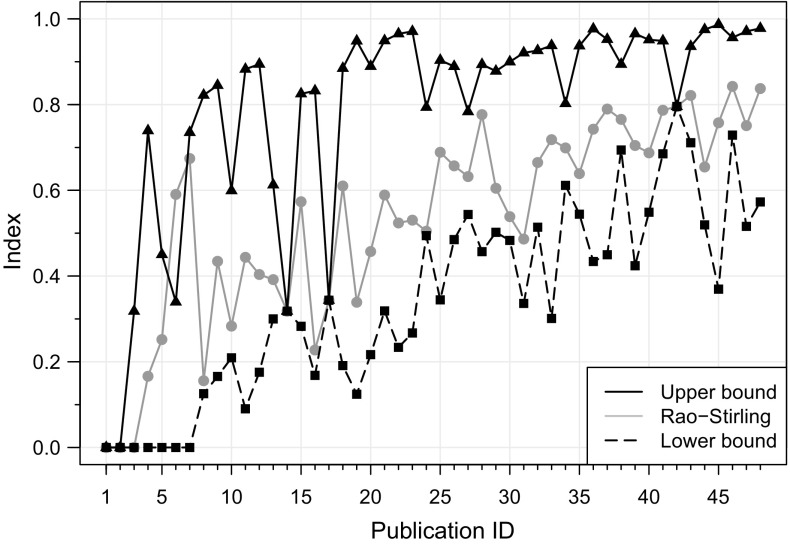
Indices of the 48 publications of the sample: Rao–Stirling calculated with completed data (*gray solid line*), upper (*black solid line*) and lower (*black dashed line*) bounds of the uncertainty interval calculated with incomplete data and parameters ${k = 4}$ and ${t = 0.233}$. The uncertainty interval includes in its range the results of the Rao–Stirling index with completed data in almost all cases, which indicates its good performance

Since the bounds of the uncertainty interval are an estimation of the possible highest and lowest Rao–Stirling index of a publication with incomplete data, its result is correct when the interval includes the Rao–Stirling index with completed data (see Fig. 4). The accuracy of the uncertainty interval is affected by the degree of categorized reference completeness of the publications.

In order to assess the performance of both our method and the Rao–Stirling index, where both use incomplete data, we compare the average of their results to the ones of the Rao–Stirling index with completed data (see Table 2). Since our method provides a measure of uncertainty, we also assess its performance by weighting the results of the uncertainty interval according to the size of the intervals, where smaller intervals have more weight than larger ones. Thus, more accurate intervals (publications with more complete data) have more weight than inaccurate intervals (publications with more incomplete data).

**Table 2:** Estimated mean, bias and standard deviation of the indices of the 48 publications of the sample: Rao–Stirling index with completed data (first row), Rao–Stirling with incomplete data (second row), the center of the uncertainty interval (third row), and the center of the uncertainty interval weighted according to its size (fourth row). These estimated values were calculated with a bootstrapped sample of 50,000 elements with replacement. A visual representation of these values can be observed in Fig. 5

Diversity index	Estimated mean	Bias	SD
Rao–Stirling with completed data	0.539	−9.646 × 10^−6^	3.308 × 10^−2^
Rao–Stirling with incomplete data	0.475	1.390 × 10^−4^	3.929 × 10^−2^
Center uncertainty interval	0.569	2.869 × 10^−5^	2.964 × 10^−2^
Weighted center uncertainty interval	0.558	1.342 × 10^−2^	3.266 × 10^−2^

**Fig. 5:**
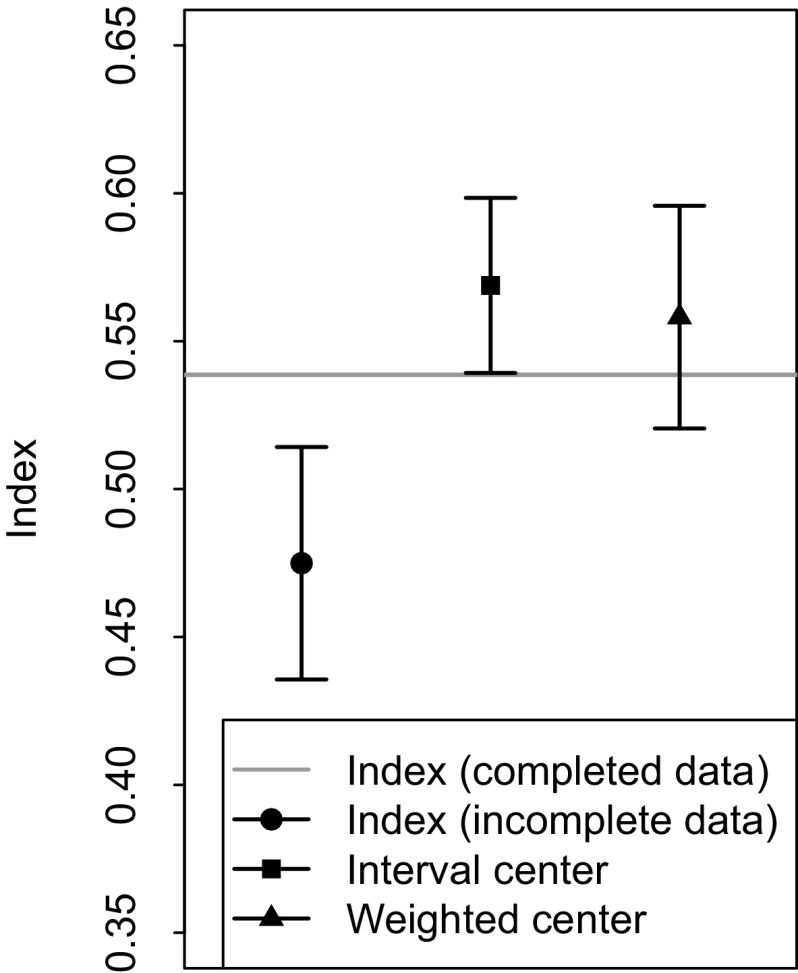
Comparison of the estimated mean of the Rao–Stirling index with completed data (*gray horizontal line*) with the estimated means and standard deviations of the Rao–Stirling index with incomplete data (*circle*), the center of the uncertainty interval (*square*) and the weighted center of the uncertainty interval (*triangle*). These estimated values were calculated with a bootstrapped sample of 50,000 elements with replacement (see Table 2). Our uncertainty interval shows a better performance than the Rao–Stirling index with incomplete data. The aggregated results of our uncertainty interval are closer to the results of the Rao–Stirling index with completed data

The results of our method are closer to the ones of the Rao–Stirling index with completed data. This suggests that the center of the uncertainty interval is a more accurate IDR measurement than the Rao–Stirling index with incomplete data. The most accurate results are those of the weighted center of the uncertainty interval, whose standard deviation even includes the actual mean of the Rao–Stirling index with completed data (see Fig. 5).

## Discussion

The accuracy of citation-based IDR measurements heavily depends on the quality of the bibliographic data. The combination of data from several sources might help to enhance the quality of data but it certainly does not assure ground-truth bibliographic data. The dataset gathered for the evaluation of our methods is an example of an incomplete one, even though data from three different digital libraries was extracted and combined. Not all publications of our dataset have a complete record of references, and not all references are categorized with CTs. The Rao–Stirling index is incapable of taking both problems into account as it is not designed to handle missing data.

Our method tackles the problem of uncategorized references, extending the Rao–Stirling index to encode the uncertainty caused by missing data as an interval. A high degree of incompleteness in publications particularly interdisciplinary in nature may also result in underestimating the upper bound of the uncertainty interval. This is especially problematic when a publication only has one reference categorized by a single CTs. Such a degree of incompleteness affects the rational redistribution of CTs needed to compute the upper endpoint of the uncertainty interval (see publication ID = 6 in Figs. 3 and 4). The main benefit of the uncertainty interval is that it acts as a confidence indicator of the results delivered by the Rao–Stirling index. On the one hand, publications with a low proportion of uncategorized references have correspondingly small uncertainty intervals, implying a more reliable measurement of the Rao–Stirling index. On the other hand, publications with a high proportion of uncategorized references have correspondingly large uncertainty intervals, indicating an unreliable measurement of the Rao–Stirling index. This finding proves the importance of selecting publications with a proportion of categorized references above a threshold value when computing an index of interdisciplinarity, as in the analysis of Rafols et al. (2012).

The empirical evaluation of our method confirms that the acknowledgment of missing data delivers a more accurate aggregated IDR measurement than the Rao–Stirling index. Our contribution constitutes a first approach to measure IDR taking into account the inaccuracy of the bibliographic data, but other problems still affect the results of the Rao–Stirling and other IDR indices. Future analysis to evaluate this method should be conducted using other taxonomies of disciplines. Further work would be needed in order to tackle the problem of incomplete and incorrect records of references, as well as incorrect categorization of publications into disciplinary fields. Additional issues to consider are the use of a precise taxonomy of disciplines and similarity matrix. Therefore, further avenues of research towards more precise IDR indicators remain open. To aid these efforts, we are providing the source code for our implementation of the uncertainty computation to the community, which can be found at https://gitlab.com/mc.calatrava.moreno/robustrao.git.

### Electronic supplementary material

Below is the link to the electronic supplementary material.

Supplementary material 1 (pdf 731 KB)

## Figures and Tables

**Fig. 6:**
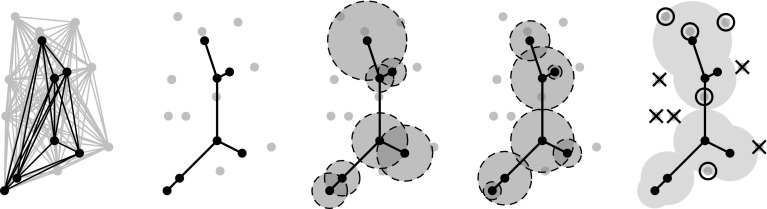
Illustration of our discipline pruning method. (*Left*) Using the similarity matrix $\mathbf {S}$ as the adjacency matrix of a complete graph, the disciplines that are known from the categorized references are represented as the vertices of the *black subgraph*. Other disciplines of $\mathcal {T}$ are given in *gray* and the similarity between two disciplines is visualized by the length of the corresponding edge, with low similarity corresponding to an increased length. (*Center left*) A spanning tree is constructed between the known disciplines, thus maximizing the mutual similarity. (*Center*) Condition *II* (see Eq. 9) ensured that each neighborhood—depicted as a ball—includes at least two disciplines. (*Center right*) Condition *I* (see Eq. 9) connects adjacent neighborhoods and guarantees the connectedness of the set of neighborhoods. (*Right*) The union of both conditions determines the final neighborhoods and all disciplines that are contained in them (*black rings*) participate in the discipline assignment. All remaining disciplines (*black cross marks*) are pruned away. Note that the actual computation takes place in a high-dimensional space and this 2D figure serves only as an illustration

## Appendix: Transformation of the optimization problem

In this section, we show how to transform the optimization problems between count space and proportion space. Starting with the optimization problem given in Eq. 2, we transform the per-discipline counts $\mathbf {n}$ into per-discipline proportions $\mathbf {p}$ by applying $\mathbf {p} = \mathbf {n}/{{||}\mathbf {n}{||}_{1}}$. The quadratic form $\frac{\mathbf {n} \, \mathbf {S} \, \mathbf {n}^\intercal }{{{||}\mathbf {n}{||}_{1}}^2}$ becomes $\mathbf {p} \, \mathbf {S} \, \mathbf {p}^\intercal$ due to linearity. The normalization of $\mathbf {p}$ is captured by the constraint ${{||}\mathbf {p}{||}_{1}} = 1$. The transformation of the other constraints requires more effort and after proving a general transformation lemma in Appendix “Constraint transformation lemma” section, we will apply it to convert the various constraints on $\mathbf {n}$ from count space to proportion space (see Appendices “Hypercube constraints” and “Constraint refinement”).

### Constraint transformation lemma

To transform the constraints given in count space to their corresponding form in proportion space, we will develop a lemma that treats the general case of arbitrary constraints. In sections Appendix we employ it to transform the concrete constraints that arise from the formulation of the discipline assignment as an optimization problem. Here and below, we represent the per-discipline count $\mathbf {n}$ as a sum of the initial count $\mathbf {c}$ and the additional count $u \mathbf {\lambda }$ that arises from the assignment of disciplines to the uncategorized references, i.e., $\mathbf {n} = \mathbf {c} + u \mathbf {\lambda }$.

#### **Lemma 1**

(Transformation Lemma) *Let*$\mathbf {q}(\mathbf {\lambda }) = \frac{\mathbf {c} + u \mathbf {\lambda }}{{{||}\mathbf {c} + u \mathbf {\lambda }{||}_{1}}} \in \mathbb {R}^n$*where*$\mathbf {\lambda } \in \mathbb {R}^n$*is contained in the intersection of the non-identical*$(n-1)$-*dimensional hyperplanes*$\mathbf {a} \cdot \mathbf {\lambda } = \alpha$*and*$\mathbf {b} \cdot \mathbf {\lambda } = \beta$. *Thus*, $\mathbf {q}(\lambda )$*constitutes a hyperplane in the space*${{||}\cdot {||}_{1}} = 1$*and the sign of the expression*

8 $$\begin{aligned} \bigl ( (u \, \beta + \mathbf {b} \cdot \mathbf {c}) \, \mathbf {a} - (u \, \alpha + \mathbf {a} \cdot \mathbf {c}) \, \mathbf {b} \bigr ) \cdot \mathbf {p} \end{aligned}$$

*determines on which side of this hyperplane a point*$\mathbf {p}$*lies*.

#### *Proof*

Since the hyperplanes intersect, we can assume—apart from $\mathbf {a} \ne \mathbf {0}$ and $\mathbf {b} \ne \mathbf {0}$—that $\mathbf {a}$ and $\mathbf {b}$ are linear independent. As a consequence, there exist $i,j \in \{1,\dots ,n\}$ with $i \ne j$ such that the elements $a_i \in \mathbf {a}$ and $b_j \in \mathbf {b}$ are nonzero. The linear system given by the hyperspace equations $\mathbf {a} \cdot \mathbf {\lambda } = \alpha$ and $\mathbf {b} \cdot \mathbf {\lambda } = \beta$ allows us to express two components of $\mathbf {\lambda }$ as

$$\begin{aligned} \lambda _i = \frac{a_j (\beta - \underline{\mathbf {b}} \cdot \underline{\mathbf {\lambda }}) - b_j (\alpha - \underline{\mathbf {a}} \cdot \underline{\mathbf {\lambda }})}{a_j b_i - a_i b_j} \quad \text {and} \quad \lambda _j = \frac{a_i (\beta - \underline{\mathbf {b}} \cdot \underline{\mathbf {\lambda }}) - b_i (\alpha - \underline{\mathbf {a}} \cdot \underline{\mathbf {\lambda }})}{a_i b_j - a_j b_i} \end{aligned}$$

where $\underline{\mathbf {v}} \in \mathbb {R}^{n-2}$ denotes a vector $\mathbf {v} \in \mathbb {R}^n$ with the *i*-th and *j*-th component removed. The components $q_m$ of $\mathbf {q}$ can be written as

$$\begin{aligned} q_m(\underline{\mathbf {\lambda }}) = \frac{1}{N(\underline{\mathbf {\lambda }})} {\left\{ \begin{array}{*{20}l} -a_j \bigl (b_i c_i + u(\beta - \underline{\mathbf {b}} \cdot \underline{\mathbf {\lambda }})\bigr ) + b_j (a_i c_i + u(\alpha - \underline{\mathbf {a}} \cdot \underline{\mathbf {\lambda }})) &{} m = i \\ -a_i \bigl (b_j c_j + u(\beta - \underline{\mathbf {b}} \cdot \underline{\mathbf {\lambda }})\bigr ) + b_i (a_j c_j + u(\alpha - \underline{\mathbf {a}} \cdot \underline{\mathbf {\lambda }})) &{} m = j \\ (a_j b_i - a_i b_j)(c_k + u \lambda _k) &{} m \ne i,j \end{array}\right. } \end{aligned}$$

with

$$\begin{aligned} N(\underline{\mathbf {\lambda }}) = u(a_j - a_i)(\beta - \underline{\mathbf {b}} \cdot \underline{\mathbf {\lambda }}) + u(b_i - b_j)(\alpha - \underline{\mathbf {a}} \cdot \underline{\mathbf {\lambda }}) + (b_i + b_j)({{||}\mathbf {c}{||}_{1}} + u {{||}\underline{\mathbf {\lambda }}{||}_{1}}). \end{aligned}$$

To compute the $n-2$ vectors that span the $(n-2)$-dimensional space of $\mathbf {q}(\underline{\mathbf {\lambda }})$, we compute its derivative with respect to all components $\lambda _k$ of $\underline{\mathbf {\lambda }}$ with $k \in \{1,\dots ,n\}$ and $k \ne i,j$. As only the sign of the final expression is of interest, uniform scaling of these vectors is permitted and we omit the $N(\underline{\mathbf {\lambda }})^{-2}$ term that arises with the differentiation. We get

$$\begin{aligned} \frac{\partial q_m}{\partial \lambda _k} = {\left\{ \begin{array}{*{20}l} u \bigl ( \alpha (b_j - b_k) + \beta (a_k - a_j) \bigr ) + (a_k b_j - a_j b_k) {{||}\mathbf {c}{||}_{1}} + D c_i &{} m = i \\ u \bigl ( \alpha (b_k - b_i) + \beta (a_i - a_k) \bigr ) + (a_i b_k - a_k b_i) {{||}\mathbf {c}{||}_{1}} + D c_j &{} m = j \\ u \bigl ( \alpha (b_i - b_j) + \beta (a_j - a_i) \bigr ) + (a_j b_i - a_i b_j) {{||}\mathbf {c}{||}_{1}} + D c_k &{} m = k \\ D c_m &{} m \ne i,j,k \end{array}\right. }, D = \left|{\begin{array}{*{20}l} a_i &{} a_j &{} a_k \\ b_i &{} b_j &{} b_k \\ 1 &{} 1 &{} 1 \end{array}}\right|. \end{aligned}$$

Together with the normal vector $\mathbf {1} = (1,\cdots ,1)$ of the ${{||}\, \cdot \,{||}_{1}} = 1$ hyperplane, of which $\mathbf {q}(\underline{\mathbf {\lambda }})$ is a subset, we can compute the ‘binormal’ vector $\mathbf {r}$ as the $(n-1)$-ary product

of the $(n-2)$ derivatives, the normal vector $\mathbf {1}$ and the set of standard basis vectors $\mathbf {e}_1, \cdots , \mathbf {e}_n$ and we obtain

$$\begin{aligned} \mathbf {r} = (\mathbf {a} \cdot \mathbf {c} + u \, \alpha )({{||}\mathbf {b}{||}_{1}} \mathbf {1} - n \, \mathbf {b}) - (\mathbf {b} \cdot \mathbf {c} + u \, \beta )({{||}\mathbf {a}{||}_{1}} \mathbf {1} - n \, \mathbf {a}). \end{aligned}$$

The scaled signed distance between an arbitrary point $\mathbf {p}$ and the hyperplane defined by its normal vector $\mathbf {r}$ yields the desired expression

$$\begin{aligned} \bigl (\mathbf {p} - \mathbf {q}(\mathbf {0})\bigr ) \cdot \mathbf {r} = \bigl ( (u \, \beta + \mathbf {b} \cdot \mathbf {c}) \, \mathbf {a} - (u \, \alpha + \mathbf {a} \cdot \mathbf {c}) \, \mathbf {b} \bigr ) \cdot \mathbf {p}. \end{aligned}$$

$$\square$$

### Hypercube constraints

In this section, we transform the constraints $c_i \le n_i \le c_i + u$ of Eq. 2, which describe a hypercube in ${N_{\mathcal {T}}}$ dimensions, to proportion space. We will abbreviate ${N_{\mathcal {T}}}$ with *N* and see that due to the normalization by the 1-norm, the hypercube is projected onto a $(N - 1)$-dimensional hyperplane along the radial directions. First, we observe that after the projection, the hypercube vertex ${\mathbf {c}}_{\text {min}}= (c_1,\ldots ,c_N)$ that lies closest to the origin is a convex combination of its neighboring vertices, i.e., for $\mathbf {\mu } = (\mu ,\ldots ,\mu _N)$ with ${{||}\mathbf {\mu }{||}_{1}} = 1$ we have that

$$\begin{aligned} \frac{{\mathbf {c}}_{\text {min}}}{{{||}{\mathbf {c}}_{\text {min}}{||}_{1}}} = \frac{\mathbf {c}}{{{||}\mathbf {c}{||}_{1}}} = \sum _{i=1}^{N} \mu _i \frac{{\mathbf {c}} + \hat{\mathbf {{u}}}_i}{{{||}\mathbf {c}{||}_{1}} + u} = \frac{\mathbf {c} + u \, \mathbf {\mu }}{{{||}\mathbf {c}{||}_{1}} + u} \implies \mathbf {\mu } = \frac{\mathbf {c}}{{{||}\mathbf {c}{||}_{1}}}, \end{aligned}$$

which confirms the convexity of the combination since $0 \le c_i / {{||}\mathbf {c}{||}_{1}} \le 1$. ${\hat{\mathbf {{u}}}}_{i}$ denotes a vector of zeros with *u* as the *i*-th component and we will use $\check{\mathbf {{u}}}_{i}$ for a vector of *u* entries with zero at the *i*-th component. The vertex ${\mathbf {c}}_{\text {max}}= (c_1 + u,\ldots ,c_N + u)$ that lies farthest from the origin can also be represented by a convex combination of its neighbors, since

$$\begin{aligned} \frac{{\mathbf {c}}_{\text {max}}}{{{||}{\mathbf {c}}_{\text {max}}{||}_{1}}}= & {} \frac{\mathbf {c} + \mathbf {u}}{{{||}\mathbf {c}{||}_{1}} + N u} = \sum _{i=1}^{N} \mu _i \frac{\mathbf {c} + \check{\mathbf {{u}}}_i}{{{||}\mathbf {c}{||}_{1}} + (N-1)u} = \frac{\mathbf {c} + \mathbf {u} - u \, \mathbf {\mu }}{{{||}\mathbf {c}{||}_{1}} + (N-1)u}\\ \implies \mathbf {\mu }= & {} \frac{\mathbf {c} + \mathbf {u}}{{{||}\mathbf {c}{||}_{1}} + N u}. \end{aligned}$$

This leads us to the conclusion that all $(n-1)$-dimensional facets that contain either ${\mathbf {c}}_{\text {min}}$ or ${\mathbf {c}}_{\text {max}}$ lie completely in the interior of the hypercube’s projection and, consequently, their $(n-2)$-facets that contain those vertices do not contribute to the boundary of the projected hypercube. Note that is not the case for any other facet. This also indicates that the constraints ${{||}\mathbf {c}{||}_{1}} + u \le {{||}\mathbf {n}{||}_{1}} \le {{||}\mathbf {c}{||}_{1}} + (N-1) \, u$ and ${{||}\mathbf {c}{||}_{1}} \le {{||}\mathbf {n}{||}_{1}} \le {{||}\mathbf {c}{||}_{1}} + N u$ are effectively equivalent after projection onto ${{||}\, \cdot \,{||}_{1}} = 1$.

To determine the form of the constraints $c_i \le n_i \le c_i + u$ in proportion space, we project the $(n-2)$-dimensional ridges of the associated hypercube onto the $(n-1)$-dimensional hyperplane defined by ${{||}\, \cdot \,{||}_{1}} = 1$. Each ridge is given as an intersection of two of the hyperplanes that contain the facets of the hypercube. For $\mathbf {n} = \mathbf {c} + u \, \mathbf {\lambda }$, they are given as $\lambda _i = 1$ and $\lambda _j = 0$. All ridges can be obtained by varying $i,j \in \{1,\ldots ,n\}$ with $i \ne j$. Note that $\mathbf {\lambda } = \mathbf {0}$ or $\mathbf {\lambda } = \mathbf {1}$ are omitted due to the convexity argument given above.

In the context of Lemma 1, the hyperplane equations are $\mathbf {a} = \hat{\mathbf {1}}_i$ and $\alpha = 1$ as well as $\mathbf {b} = \hat{\mathbf {1}}_j$ and $\beta = 0$. An application of Eq. 8 gives the expression $(u + c_i)p_j - c_j p_i \ge 0$ as criterion that $\mathbf {p}$ lies inside the projection of the hypercube. This gives the hypercube constraints of the optimization problem, as stated in Eq. 2, in proportion space (see Eq. 3) as

$$\begin{aligned} p_i \le \frac{c_i + u}{c_j} p_j, \quad i,j \in \{1,\ldots ,N\}. \end{aligned}$$

Note that these constraints trivially hold for the diagonal elements $i=j$ as well, since $u \ge 0$. In the case of vanishing reference count $c_j$ for a given discipline *j*, we simply set $p_i \le \infty$ and effectively omit the constraint.

### Constraint refinement

By limiting the number of disciplines that each uncategorized reference can be assigned to by *k*, we arrive at the additional count-space constraint ${{||}\mathbf {n}{||}_{1}} \le {{||}\mathbf {c}{||}_{1}} + k \, u$ (see Eq. 4). With the hypercube constraints of the previous section and after writing $\mathbf {n} = \mathbf {c} + u \, \mathbf {\lambda }$, it can be stated as ${{||}\mathbf {\lambda }{||}_{1}} = k$ and $\lambda _i \ge 0, i \in \{1,\ldots ,N\}$. In terms of Lemma 1, we have that $\mathbf {a} = \mathbf {1}$ and $\alpha = k$ as well as $\mathbf {b} = \hat{\mathbf {1}}_i$ and $\beta = 0$. Applying the term given by Eq. 8 yields $-c_i {{||}\mathbf {p}{||}_{1}} + (u \, k + {{||}\mathbf {c}{||}_{1}}) p_i \ge 0$ and we obtain the proportion-space equivalent of the constraint in Eq. 4 as

$$\begin{aligned} p_i \ge \frac{c_i}{{{||}\mathbf {c}{||}_{1}} + k \, u}, \quad i \in \{1,\ldots ,N\}. \end{aligned}$$

## Discipline pruning

In this section, we present a method to compute the set ${\mathcal {T}}_{\text {valid}}$ of disciplines that fulfills the requirements laid out in “Discipline pruning” section with an accompanying illustration given in Fig. 6. For this, we exploit the properties of the similarity matrix $\mathbf {S}$ that encodes the closeness between different scientific disciplines. Since it is symmetric (i.e., discipline $\tau _i$ has the same similarity with $\tau _j$ as $\tau _j$ with $\tau _i$) and its entries are non-negative, it can be seen as the adjacency matrix of a *complete* undirected graph $\mathcal {G}$ with positive (or vanishing) edge weights. Note that while the conventional similarity matrix has ones along its diagonal, we assume that graph to be loop-free without invalidating our argument. We will denote the set of vertices of a graph *g* with *V*(*g*) and its edges with *E*(*g*), where the latter is a subset of $V(g) \times V(g)$.

Each vertex in $V(\mathcal {G})$ corresponds to a discipline, whereas an edge in $E(\mathcal {G})$ with non-zero weights indicates a certain similarity between its respective disciplines. We now construct the set ${\mathcal {T}}_{\text {valid}}$ of vertices and later validate it against the required properties of “Discipline pruning” section. In the first step, we take the subgraph ${\mathcal {G}}_{\text {known}}$ of $\mathcal {G}$ that contains as vertices only those disciplines that are found in the categorized references of the document at hand, which yields again a complete graph. Next, a *maximum spanning tree* ${\mathcal {G}}_{\text {span}}$ is computed from ${\mathcal {G}}_{\text {known}}$ and for each of its vertices $v_i \in V(\mathcal {G}_{\text {span}})$, we compute a *local similarity threshold* $\Delta _i$ by

9 $$\begin{aligned} \Delta _i= & {} t \min \bigg ( \underbrace{ \min \big \{ 2 w(e) : e \in E({\mathcal {G}}_{\text {span}}) {\text { and }} v_i \in e \big \} }_{I},\nonumber \\&\underbrace{ \max \big \{ w(e) : e \in E({\mathcal {G}}_{\text {span}}) {\text { and }} v_i \in e \big \} }_{II} \bigg ) \end{aligned}$$

where the weight of an edge *e* is denoted by *w*(*e*) and *t* is a user-given tolerance value in the interval [0, 1]. In the final step, we construct around each vertex $v_i$ of the spanning tree ${\mathcal {G}}_{\text {span}}$ a discipline neighborhood $\mathcal {H}_i$ given by

$$\begin{aligned} {\mathcal {H}}_{i} = \{ v_j \in V({\mathcal {G}}) : w(e_{ij}) \ge \Delta _i {\text { and }} \{ v_i, v_j \} \in {e_{ij}} \} \end{aligned}$$

where $e_{ij}$ is an edge of the initial complete graph $\mathcal {G}$ that contains both vertices $v_i$ and $v_j$. The set ${\mathcal {T}}_{\text {valid}}$ of disciplines that participate in the reassignment process are obtained by the union of all neighborhoods, i.e.,

$$\begin{aligned} {\mathcal {T}}_{\text {valid}}= \bigcup _{v_i \in V({\mathcal {G}}_{\text {span}})} {\mathcal {H}}_i. \end{aligned}$$

This definition of ${\mathcal {T}}_{\text {valid}}$ fulfills all objectives stated in “Discipline pruning” section for a tolerance of $t = 1$:

*Completeness*: Due to condition II in the computation of $\Delta _i$, each neighborhood $\mathcal {H}_i$ contains at least one edge of $E(\mathcal {G})$ and, consequently, its two endpoints.
*Cohesion*: Due to condition I in the computation of $\Delta _i$ and the fact that a spanning tree of a complete graph is connected, the neighborhoods form a single connected set.
*Conciseness*: The maximal spanning tree ${\mathcal {G}}_{\text {span}}$ is the subgraph with the highest internal similarity that still provides a connected subgraph. In this sense, it produces the smallest neighborhoods that are still connected due to the fact that condition I ensures that the neighborhoods only ‘touch’ along the edge with the least similarity.

Note that higher tolerances ($t < 1$) violate these objectives only in their original sense but would respect them for appropriately scaled similarity values.
