# Supplementary material for: On the uncertainty of interdisciplinarity measurements due to incomplete bibliographic data
Source: Scientometrics. 2016 Feb 9;107:213–32. doi: 10.1007/s11192-016-1842-4 (PMC4819562; doi:10.1007/s11192-016-1842-4)
Supplement: Supplementary file 1 — Supplementary material 1 (pdf 731 KB) [file 11192_2016_1842_MOESM1_ESM.pdf]

On the Uncertainty of Interdisciplinarity Measurements Due to Incomplete Bibliographic Data

Cover Letter Supplementary

Abstract

This document provides an example of the NP-hardness of reference redistribution as described in Section 3.6 (Pardalos and Vavasis 1991; Sahni 1974). It serves as an illustration of the fact that a redistribution of all uncategorized references to the discipline with the highest number of categorized references does not yield the minimal diversity index in general.

Introduction

Since the similarity matrix *S* that is used in the Rao-Stirling diversity index is positive semidefinite, the index, given by

In[1]:=
```
Index[c_, S_] := 1 - c.S.c^T / (Total[c, 2])^2 // FullSimplify // #[[1, 1]] &
```

$$\text{Index}[c\_, S\_] := 1 - \frac{c.S.c^{T}}{(\text{Total}[c, 2])^{2}} \;//\; \text{FullSimplify} \;//\; \#[[1, 1]] \;\&$$

is a concave function in *c*. As described in Section 3, the minimization of this function is used to compute the lower bound of the uncertainty interval. Due to the purely concaveness, the minima lie on the vertices of the polytope that is spanned by the constraints on *c* (Floudas and Visweswaran 1995).

Example Setting

We provide a simplified setting to show the complexity of the minimization problem. If we assume only the existence of three different disciplines, the similarity matrix can be given as

In[2]:=

$$\text{Similarity}[\alpha\_] := \begin{pmatrix} 1 & \alpha & 0 \\ \alpha & 1 & 0 \\ 0 & 0 & 1 \end{pmatrix}$$

where α describes the similarity between the first and the second disciplines. We assume that the third discipline is completely dissimilar to the first and second discipline.

A hypothetic document with both categorized and uncategorized references serves as the basis for this

example. We assume the following categorized references in the three disciplines

In[3]:=
```
categorized[c3_] := ( 2  2  c3 )
```

where the first two disciplines are cited by two references each. The number of references that cite the third discipline is given by c3. Finally, the number of uncategorized references is given by *u*. In total $2 + 2 + c3 + u$ references are present in this example of which *u* references are uncategorized.

Example Minimization

The diversity index of our setting is thus given as

In[4]:=
```
Index[categorized[c3] + u ( λ  μ  1 - λ - μ ), Similarity[α]]
```

Out[4]=

$$\frac{1}{(4 + c3 + u)^2} 2 \left(4 - 4\alpha - 2u(-2 + \lambda + \alpha\lambda + \mu + \alpha\mu) - u^2\left((-1 + \lambda)\lambda + (-1 + \lambda + \alpha\lambda)\mu + \mu^2\right) + c3\,(4 + u(\lambda + \mu))\right)$$

where $0 \le \lambda \le 1$ (resp. $0 \le \mu \le 1 - \lambda$) determine the extent at which the uncategorized references are redistributed to the first (resp. second) discipline. The remainder is redistributed to the third discipline. Since the minimum of the index is attained at the vertices of the constraint polytope, it can only be located at one of three extremal reference redistributions I1, I2, and I3. For these, all the uncategorized references are redistributed to the either the first, second, or third discipline. The diversity index of these three extremal cases is given as

In[5]:=
```
I1[α_, c3_, u_] := Index[categorized[c3] + u ( 1  0  0 ), Similarity[α]]
I2[α_, c3_, u_] := Index[categorized[c3] + u ( 0  1  0 ), Similarity[α]]
I3[α_, c3_, u_] := Index[categorized[c3] + u ( 0  0  1 ), Similarity[α]]
```

For a fixed similarity between the first and second discipline of $\alpha = 0.6$, these quanities can be plotted as surfaces over the number of uncategorized references *u* and the number of references c3 that cite the third discipline.

In[8]:=
```
Module[{α = 0.6},
 Plot3D[{I1[α, c3, u], I3[α, c3, u]}, {c3, 0, 6},
  {u, 1, 5}, AxesLabel → {c3, u}, PlotLegends → {I1, I3}]]
```

Out[8]=

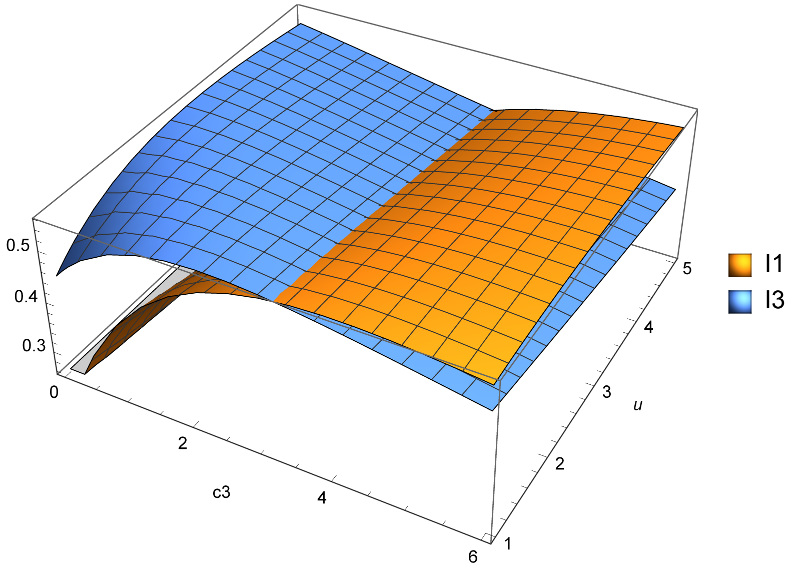

To clarify the exposition, we chose a fixed $u = 2$ for the following discussion. Note that the same conclusion is valid for any $u > 1$.

In[9]:=
```
Module[{α = 0.6, u = 2},
 Plot[{I1[α, c3, u], I3[α, c3, u]}, {c3, 0, 5},
  PlotLegends → {I1, I3}, PlotTheme → "Detailed", FrameLabel → c3]
]
```

Out[9]=

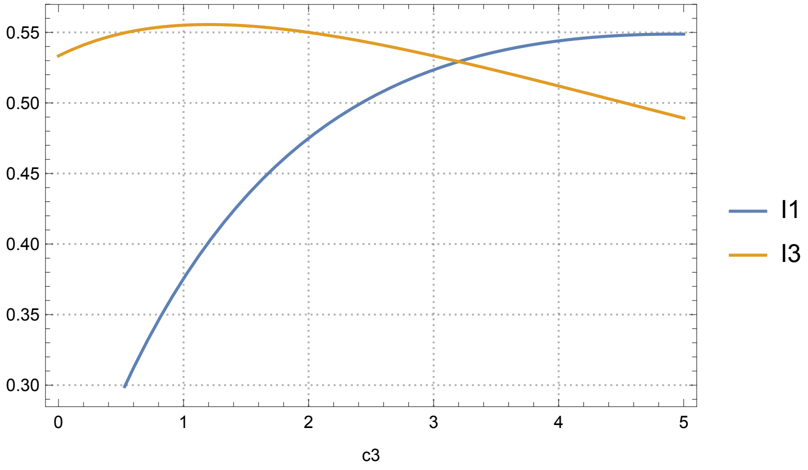

Depending on the value of c3, the minimum can be attained by redistributing all references to either the first or third discipline. In the case of c3 = 3, the minimum can be achieved by redistributing the uncategorized references to the first discipline (i.e., I1 < I3 for c3 = 3). For c3 = 4, a redistribution to the third discipline yields the minimal index (i.e., I3 < I1 for c3 = 4).

Conclusion

Thus, it cannot be assumed that simply adding all uncategorized references to the discipline with the highest number of categorized references would yield the minimal index. In **both** afore-mentioned cases, the third discipline has the highest number of categorized references ($c3 = 3$, $4 > 2$).
